# Extranodal relapse of anaplastic large cell lymphoma around the Achilles tendon masquerading as a posttraumatic hematoma

**DOI:** 10.1002/jha2.293

**Published:** 2021-09-12

**Authors:** Kajal Joshi, Ruqaiya Abdul Samad, Dipali Jesrani, Rehmat Solanki, Wassan Al Saedi, David Dennison

**Affiliations:** ^1^ General Practice Apollo Hospital Oman Muscat Oman; ^2^ Department of Radiology Apollo Hospital Oman Muscat Oman; ^3^ Department of Hematology Apollo Hospital Oman Muscat Oman

An 11‐year‐old girl with cervical node ALK+ anaplastic large cell lymphoma (ALCL) in remission for 3 years after chemotherapy complained of dull ache in the lower part of both legs just above the heels. About 2–3 months later she sustained an accidental blunt injury to the lower part of the right (R) leg for which her parents sought medical attention. On examination, there was a fluctuant but tense swelling in the posterior aspect of the R lower leg (Figure 1). There was no warmth and ankle movements were normal. There was no significant lymph node enlargement and the liver or spleen was not palpable. A hematoma was suspected. The complete blood count and blood film were unremarkable. However, magnetic resonance imaging (MRI) revealed a well‐defined lobulated lesion centered at the pre‐Achilles fat pad, with central necrosis and encasing, but not involving, the Achilles tendon (Figure 2). In one area, the lesion was seen to cross the subcutaneous fat, entering the skin (correlating with Figure 1) through a narrow opening lateral to the Achilles tendon. Muscle or bone was not involved. Histopathology of the biopsied tissue showed a diffuse infiltrate of lymphoid cells with typical pleomorphic morphology of ALCL, horse shoe nuclei, frequent mitosis, and increased vascularity (Figure 3). Immunohistochemistry was positive for CD3, CD8, CD30, ALK 1, and Ki‐67 (70%), consistent with relapsed ALCL.

**FIGURE 1:**
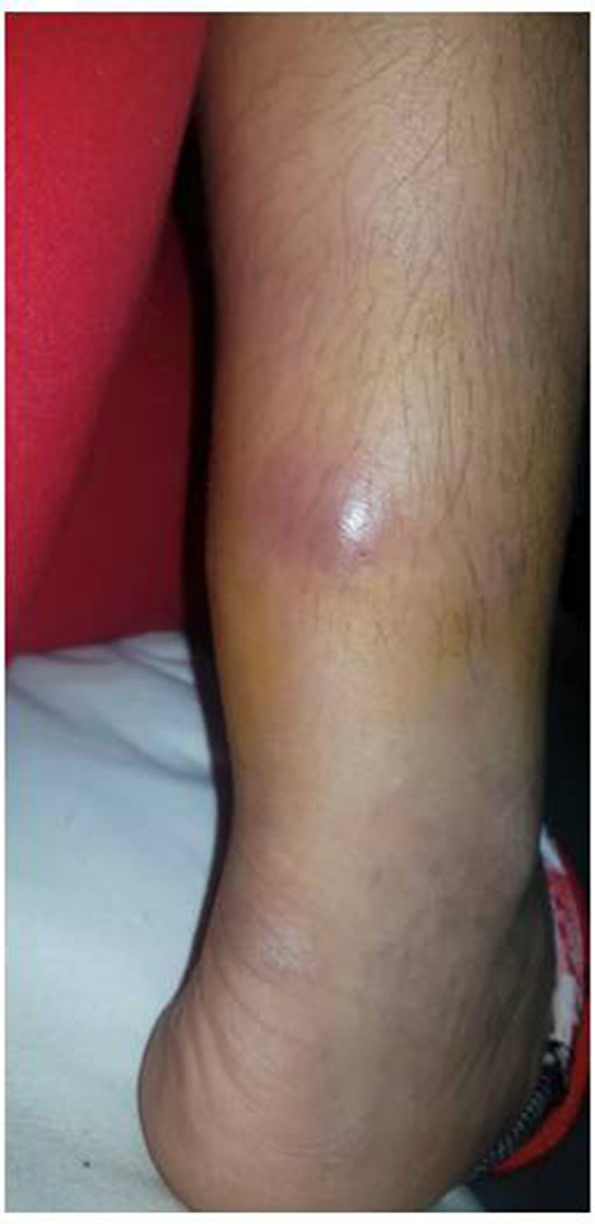
Clinical picture of leg at presentation

**FIGURE 2:**
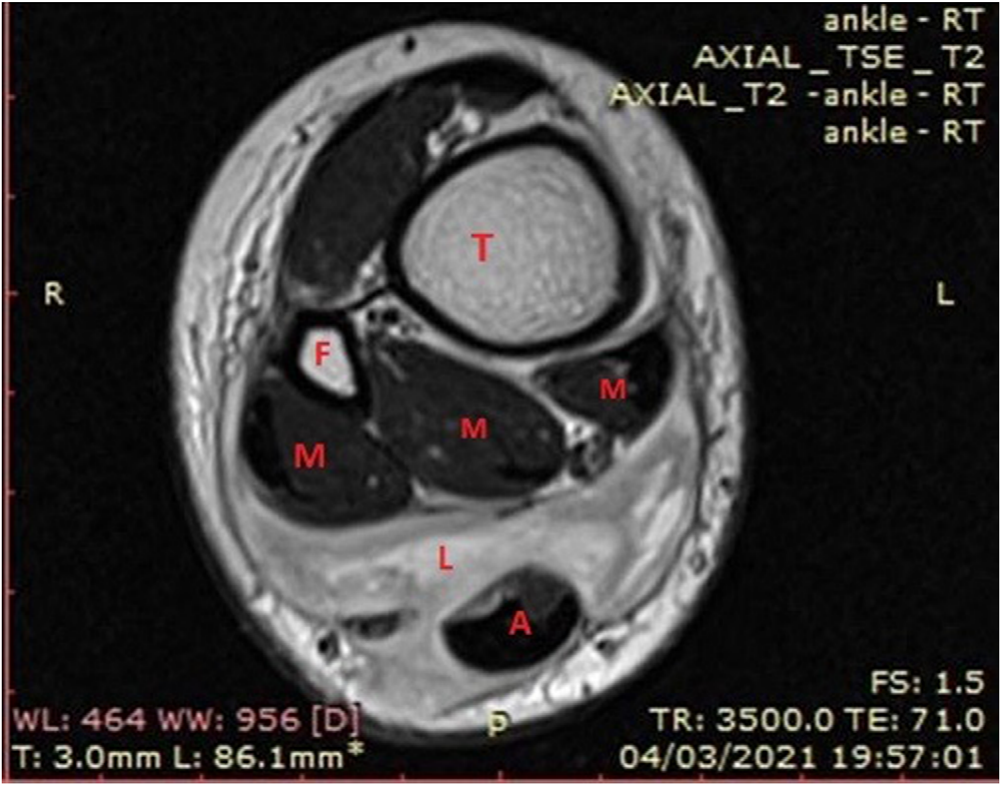
MRI R lower limb: T = tibia, F = fibula, M = muscle, L = lymphoma, A = Achilles tendon

**FIGURE 3:**
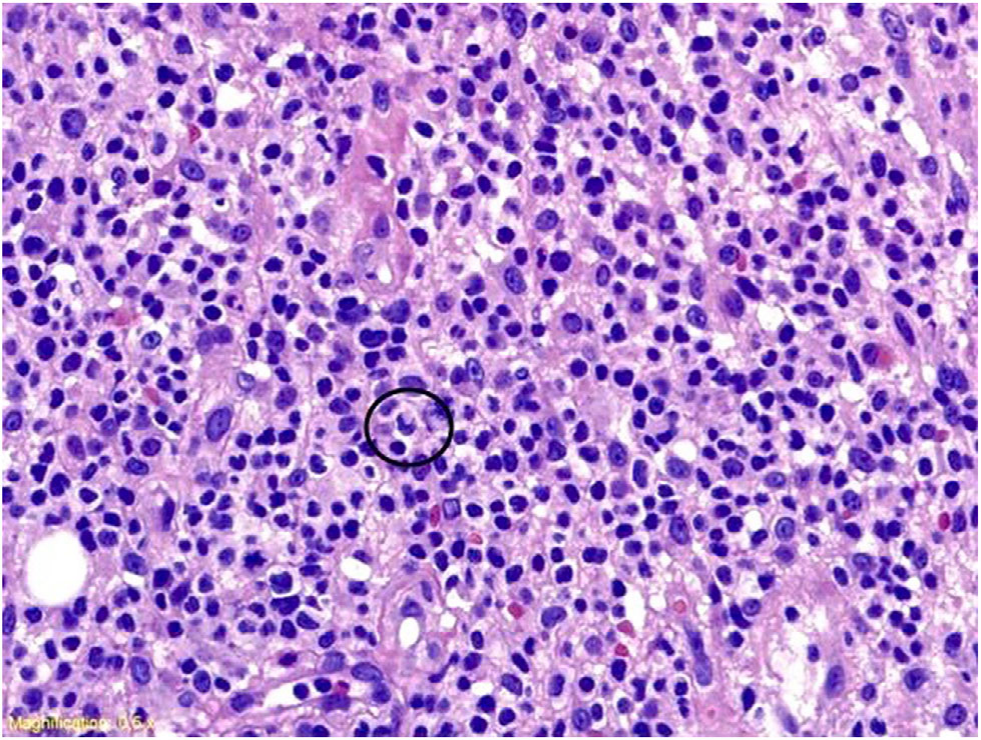
Histopathology of biopsy: Circled area shows cell with horseshoe‐shaped nucleus

We would like to highlight this unusual site of extranodal relapse of ALCL, occurring in the soft tissue surrounding the Achilles tendon and masquerading as a posttraumatic hematoma.

## CONFLICT OF INTEREST

The authors declare no conflict of interest.

